# Copper (II) Ions Induced Self-Disproportionation of Enantiomers in Capillary Electrophoresis for the Quantification of Atenolol Enantiomers

**DOI:** 10.3390/molecules28155908

**Published:** 2023-08-06

**Authors:** Shaoqiang Hu

**Affiliations:** Henan Key Laboratory of Function-Oriented Porous Materials, College of Chemistry and Chemical Engineering, Luoyang Normal University, Luoyang 471934, China; shaoqianghu@lynu.edu.cn

**Keywords:** chiral separation, capillary electrophoresis (CE), self-disproportionation of enantiomers (SDE), ligand exchange, atenolol

## Abstract

Despite the fact that the self-disproportionation of enantiomers (SDE) has been found for several decades and has been widely used in crystallization, sublimation and chromatography for the purification or separation of nonracemic compounds, the phenomenon of SDE in capillary electrophoresis (CE) has never been reported up to now. Here, a new approach to separate enantiomers in CE based on SDE was demonstrated by introducing copper (II) ions into the separation media. The enantiomers of atenolol interact with copper ions to produce positively charged complexes with different electrophoretic mobilities from the single molecules. The dynamic equilibrium between homo- or heterochiral complexes (associates) and single molecules of atenolol enantiomers supports the manifestation of SDE. Different mobilities of the single molecules and associates, and different distribution of two enantiomers between the single molecules and associates caused by their different concentrations, produce a net difference in electrodriven migration velocities of the two enantiomers. The relative movement of two enantiomers causes a zone depleted in one enantiomer at the rear end of sample segment, giving a trapezoidal CE curve with a step at the end. Quantification of enantiomers is achieved according to the step height. The analysis does not rely on the use of enantiomerically pure chiral selector and the result agrees with that obtained by conventional chiral CE using a chiral selector.

## 1. Introduction

Chiral analysis to determine the enantiomeric purity or the relative ratio of enantiomers is necessary in the asymmetric synthesis of chiral compounds and pharmaceutics as enantiomers often differ in their biological activities and binding properties [1,2,3,4]. This is most commonly achieved by using a chiral selector to form diastereomeric complexes with the enantiomers that lead to their separation [5,6,7,8]. Although a large number of chiral selectors including cyclodextrins, crown ethers, macrocyclic antibiotics, chiral surfactants, chiral ionic liquids, chiral nanomaterials, chiral membranes and metal-organic frameworks have been studied and applied in the separation of different types of chiral compounds [9,10,11,12,13,14], the analysis of some chiral drugs still faces some challenges, including the narrow choice of available chiral selectors [15,16].

The self-disproportionation of enantiomers (SDE) provides another mechanism for the separation of enantiomers by nonconventional techniques, not relying on a chiral selector. The SDE refers to the spontaneous fractionation of nonracemic material into enantioenriched and depleted fractions when any physicochemical process is applied under totally achiral conditions [17,18,19]. The purification or separation of nonracemic sample by fractional crystallization and sublimation based on the SDE principle can be recalled from very early times, and is still of interest for many researchers [20,21,22,23]. Additionally, since the seminal works of P. A. Crooks [24] and E. Gil-Av [25], the SDE on different types of achiral chromatography including routine, gravity-driven column chromatography, medium pressure liquid chromatography (MPLC), high-performance liquid chromatography (HPLC) and gas chromatography (GC) has been intensively studied [26,27,28,29,30,31,32]. The underlying process giving rise to the SDE is the presence or formation of homo- and heterochiral associates, or a dynamic equilibrium of single molecules with homo- and heterochiral associates [19,33,34]. The mechanism of SDE in chromatography is the rapid exchange of enantiomers between single molecules and homo- or heterochiral associates [35,36,37,38,39]. The separation by SDE is ascribed to the difference in elution rate between the single molecules and the aggregates as well as different distribution of two enantiomers between the single molecules and homo- or heterochiral associates regulated by the energy difference between the two associations and the concentrations of the two enantiomers [19,40]. By contrast, with the wide attention to SDE in chromatography, the SDE in capillary electrophoresis (CE), another high-efficiency separation technology, has never been reported [19]. This is probably because aqueous solutions and other polar solutions are usually employed in CE as background electrolyte (BGE). An effective analyte–analyte intermolecular interaction to produce associates as the base of SDE is excluded or extremely limited in the separation media by the strong analyte–solvent intermolecular interaction. Therefore, it is difficult to find the SDE in CE analysis.

Ligand exchange (LE) is an important approach to resolving enantiomers in conventional chiral separation [41,42,43,44]. It has been widely used in CE (LE-CE) for the enantioseparation of amino acids and other chelating compounds with amino, hydroxyl, or carboxyl groups by using a chiral ligand metal complex as chiral selector [45,46,47,48,49]. The separation mechanism is based on the formation of diastereomeric ternary mixed metal complexes between the enantiomerically pure selector ligand and the analyte:

M (L-selector)_2_ + *S*-analyte ⇌ (L-selector) M (*S*-analyte) + (L-selector) (1)

M (L-selector)_2_ + *R*-analyte ⇌ (L-selector) M (*R*-analyte) + (L-selector) (2)

The different thermodynamic stability of the mixed complexes between two enantiomers as well as different mobility of the analyte and complex are responsible for the enantioseparation [41,42,43,44]. Here, an enantiomerically pure chiral selector remains an indispensable factor for the separation of enantiomers.

Inspired by the mode of LE-CE, a new approach to achieve the test of SDE in CE, by using central ions to bridge the association of analyte through the formation of complex, was proposed in this work. Copper (II) ions were added into the BGE containing ammonia (0.2 M, pH 11.0). Atenolol (Ate), a chelate-forming β-blocker and an important chiral drug, was selected as a model analyte. It reacts with copper ions to produce mono- or binuclear complexes of 1:2 or 2:2 (Cu:Ate) [50,51,52] with different electrophoretic mobilities from the single molecules. So, the homo- or heterochiral associates of atenolol enantiomers were formed with a dynamic equilibrium with the single molecules, supporting the manifestation of the SDE.

## 2. Principle and Protocol of Quantification

Atenolol is a basic drug with an amino group in molecule and a pKa of 9.67 (data of protonated ions from the Chemicalize). Therefore, the state of single molecules in the BGE of pH 11.0 mainly exists as neutral molecules with a very low effective electrophoretic mobility close to zero. With a stronger complexation with metal ions than ammonia, they replace the weak ligands and combine with copper ions to produce positively charged mono- or binuclear complexes with a pronouncedly bigger mobility than neutral single molecules. Given the two enantiomers of atenolol, three equilibria exist:

2 *S*-Ate + xCu(II) ⇌ (*S*-Ate) Cu(II)_x_ (*S*-Ate) (3)

*S*-Ate + *R*-Ate + xCu(II) ⇌ (*S*-Ate) Cu(II)_x_ (*R*-Ate) (4)

2 *R*-Ate + xCu(II) ⇌ (*R*-Ate) Cu(II)_x_ (*R*-Ate) (5)

where x equals 1 or 2.

For racemic mixtures containing two enantiomers of different concentrations (e.g., *C*_S_ > *C*_R_), the *S*-enantiomer will form a complex containing primarily only *S*-atenolol via reaction (3) while the *R*-enantiomer will mainly form a mixed complex via reaction (4), because of the effect of high concentration of *S*-enantiomer on the equilibrium. Only a small part of the *R*-enantiomer forms the enantiomerically pure complex via reaction (5); suppose the equilibria constant of reaction (3) or (5) is not much bigger than that of reaction (4). Therefore, the *R*-enantiomer will have a bigger proportion in the copper complex than the *S*-enantiomer. This means that the different distributions of two enantiomers between single molecules and homo- or heterochiral associates would be caused by their different concentrations. Electrodriven migration only occurs when the atenolol is in the Cu(II) complex; thus, the *R*-enantiomer will have a bigger effective mobility than the *S*-enantiomer. This leads to different velocities of the two enantiomers.

**Figure 1:**
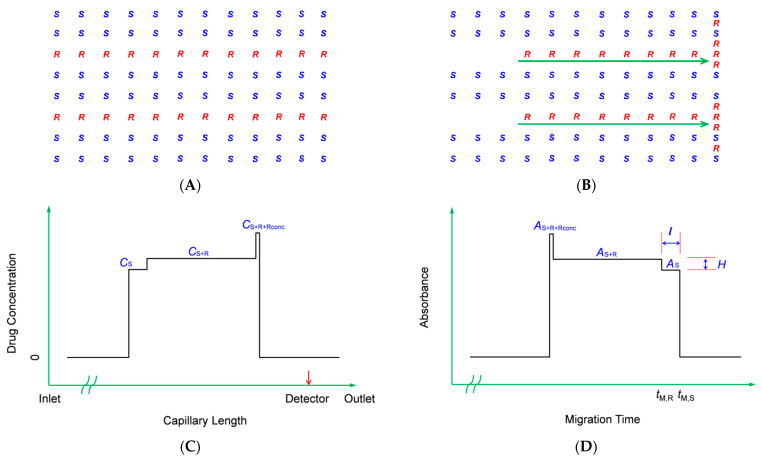
Schematic representation of the separation mechanism. (**A**). Sample segment at the beginning with higher concentration of *S*-atenolol than *R*-atenolol; (**B**). The *R*-enantiomer shifts toward the outlet with a velocity bigger than the *S*-enantiomer and concentrates at the frontier; (**C**). Theoretical concentration distribution of atenolol enantiomers after running for a period of time; (**D**). Theoretical CE curve neglecting the diffusion of drug at boundaries. *C*s is the concentration of *S*-atenolol left at the rear end, *C*_S+R_ is the concentration of the middle zone containing *S*- and *R*-atenolol with the same concentrations as injected, and *C*_S+R+Rconc_ is the concentration at frontier containing *S*- and *R*-atenolol with concentrations as injected plus the *R*-atenolol concentrated during separation. *A*_S+R+Rconc_, *A*_S+R_ and *A*_S_ are the absorbance of atenolol enantiomers at the frontier, middle zone and rear end of sample segment, respectively. *l* and *H* are the length and the height of the step on CE curve, respectively. In addition, *t*_M,R_ and *t*_M,S_ are the migration times of *R*- and *S*-atenolol, respectively.

Since the SDE only occurs in the area of sample itself, a relatively long sample segment compared to conventional CE analysis is injected in order to obtain a persistent velocity difference between two enantiomers (Figure 1A). This causes the *R*-enantiomer to move to the leading edge of the sample segment where it would concentrate while a zone depleted in *R*-enantiomer will appear at the rear end of the sample segment (Figure 1B), theoretically providing a concentration distribution as shown in Figure 1C and an electropherogram as shown in Figure 1D. The extent of the two enantiomers could be determined by the change in height (*H*) of the CE curve.

## 3. Results and Discussion

### 3.1. Optimization of Conditions

An experimental examination of the above approach was undertaken by preparing samples with a fixed total concentration of two enantiomers but a different percentage of *R*-atenolol. In these experiments, the expected peak at the front caused by the concentrating of *R*-enantiomer never appeared clearly, possibly due to the diffusion of enantiomers at the boundary. However, a step at the end of the trapezoidal peak caused by the depletion of *R*-enantiomer was observed in the preliminary experiment.

To enhance the visibility of the enantiomeric step, the conditions were optimized. First, the concentration of Cu(NH_3_)_4_^2+^ was varied from 3.0 to 5.0 mM with the free ammonia concentration fixed at 0.2 M and the pH fixed at 11.0. The sample solution has a total drug concentration of 0.4 mg∙mL^−1^ (1.5 mM) with an *R*-atenolol content of 10.0% (m/m). It was found that when 4.0 mM Cu(NH_3_)_4_^2+^ was used, the step was slightly larger and more obvious compared to using 3.0 mM of Cu(NH_3_)_4_^2+^ (Figure S1). When the Cu(NH_3_)_4_^2+^ concentration was raised from 4.0 to 5.0 mM, the step did not change considerably but the analysis time was prolonged due to the reduction of the electroosmotic flow (EOF). This may be attributed to the adsorption of more divalent copper cations on the inner surface of capillary [53]. So, 4.0 mM Cu(NH_3_)_4_^2+^ was used in the following experiments.

Next, the effect of total drug concentration was investigated from 0.2 to 1.0 mg∙mL^−1^ (0.75 to 3.75 mM) with the *R*-enantiomer content fixed at 10.0% (m/m). As shown in Figure S2, no obvious step appears when the drug concentration is 0.2 mg∙mL^−1^, and the step becomes more obvious when the drug concentration is increased from 0.4 to 1.0 mg∙mL^−1^. Clear steps can be seen when the drug concentration reaches 0.8 mg∙mL^−1^. We believe that this is because the constant Cu(NH_3_)_4_^2+^ concentration becomes lower relative to higher drug concentrations in reaction (4). Therefore, a high concentration of *S*-enantiomer would have a higher impact on the conversion ratio of *R*-enantiomer, thus giving a bigger mobility difference between two enantiomers compared to lower drug concentrations. What presents in the electropherogram is a longer step at the end of the peak. In addition, a higher drug concentration causes a higher electroconductivity of the sample solution and thus a bigger conductivity difference between the sample band and BGE. This type of mismatch gives rise to a backward tilt of the peak and hence a steep back edge of the CE curve, providing a more obvious step at the end (Figure S2). So, 1.0 mg∙mL^−1^ was selected as the optimum total drug concentration.

### 3.2. Analytical Characteristics

**Figure 2:**
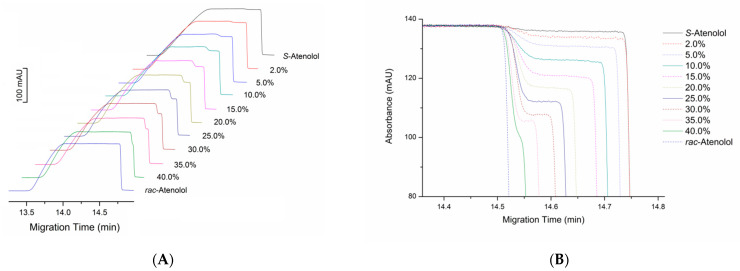
Electropherograms of the mixtures of atenolol enantiomers at optimized conditions. BGE: 4.0 mM Cu(NH_3_)_4_^2+^ in 0.2 M NH_4_OH, pH 11.0. Sample solutions: mixtures of *S*-atenolol and *R*-atenolol in BGE with a constant total concentration of 1.0 mg∙mL^−1^ while *R*-atenolol content (m/m) varied from 0 (*S*-atenolol standard) to 50% (*rac*-atenolol). Capillary: id 50 μm, od 350 μm, length 60.0 cm, 51.5 cm to detector. Capillary temperature: 20 °C. Hydrodynamic injection at 50 mBar for 60 s. Separation voltage: 30 kV. Detection wavelength: 230 nm. (**A**). Original electropherograms for samples with different *R*-atenolol contents; (**B**). Overlap of the point where the signal begins to decline for the comparison of step heights and lengths.

At optimum conditions, a clear step at the end of the trapezoidal peak was observed with a height difference (*H*) proportional to the *R*-enantiomer content (*C*) in the sample solution (Figure 2A,B), providing a regression equation of *H* = 0.9988*C* + 1.6855 with a good correlation from 0 to 30.0% (m/m) (Figure S3). This calibration was used to calculate the *R*-enantiomer content in prepared mixtures of atenolol enantiomers. Good precision and accuracy were obtained (Table 1).

**Figure 3:**
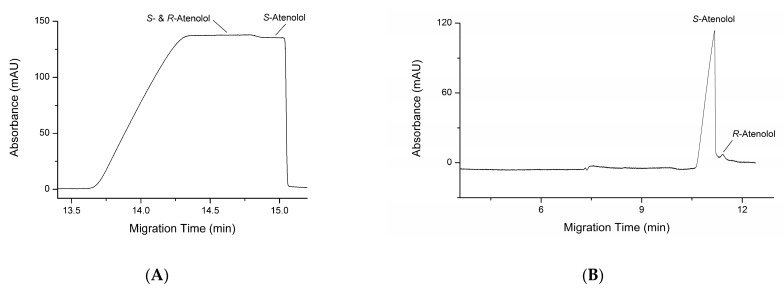
Electropherograms of *S*-atenolol standard obtained by Cu(II) ions induced SDE (**A**) and traditional chiral CE using a chiral selector (**B**). (**A**). BGE and CE conditions are the same as in Figure 2. Sample solution: 1.0 mg∙mL^−1^ *S*-atenolol in BGE. (**B**). BGE: 5.0 mM β-CDCu(II)_2_, pH 11.6. Sample solution: 0.1 mg∙mL^−1^ *S*-atenolol in BGE. Capillary: id 50 μm, od 350 μm, length 50.0 cm, 41.5 cm to detector. Capillary temperature: 20 °C. Hydrodynamic injection at 10 mBar for 20 s. Separation voltage: 15 kV. Detection wavelength: 200 nm.

It can also be seen in Figure 2A,B that a very low step appears on the CE curve of the blank solution prepared with *S*-atenolol standard, suggesting that it contains a small amount of *R*-atenolol. The content of *R*-atenolol impurity was measured with the new method developed in this work and the result was compared with the traditionally used chiral CE method using β-CDCu(II)_2_ complex as chiral selector (Figure 3). Calculated from the step height in the SDE method, the *R*-atenolol impurity is 1.69 ± 0.13% (m/m) in the *S*-atenolol standard. The result is in excellent agreement with the 1.60 ± 0.12% (m/m) calculated from the peak area in traditional chiral CE method using a chiral selector. It is slightly higher than the impurity amount in the specification of *S*-atenolol standard (HPLC purity ≥ 98.5%). The inconsistency could be attributed to the experimental errors or the configuration change of small amount enantiomers during the storage in solutions.

The pronounced advantage of the new methodology based on SDE is not relying on the use of enantiomerically pure chiral selector. This means that it is not only economical but also environmentally friendly, in line with the concept of green analytical chemistry, because most chiral selectors are synthesized by organic reactions which may produce pollutants. In addition, it has the common advantages of CE methods, such as low energy, solvent and time consuming, being the same as the traditionally used chiral CE methods. However, as a long sample segment has to be injected in the SDE method, it gives broad peaks and increases the chance of peak overlap. Therefore, it is easy for the analysis to be interfered with by other components in a sample solution and thus the robustness may be inferior compared to the traditionally used methods. In addition, since the height of a very low step on the CE curve cannot be measured accurately, it is difficult to analyze trace impurities by the SDE method. The lowest enantiomeric impurity level that can be detected is about 1% (m/m). These limitations might be overcome by further studies in the future. 

### 3.3. Confirmation of Mechanism

In order to elucidate the principle of this method, the effective mobilities of *S*- and *rac*-atenolol in different BGEs were measured (Table 2). It was found that in a BGE only containing ammonia (0.2 M, pH 11.0 adjusted with HCl) without copper ions added, the mobilities of both *S*- and *rac*-atenolol are very low due to low ionization (<5%, calculated with a pKa of 9.67). Their mobilities rise by more than four times when 3.0 mM Cu(NH_3_)_4_^2+^ is introduced, with the free ammonia concentration and the pH kept unchanged. This suggests that the enantiomers form positively charged complexes with the copper ion by the replacement of ammonia. The mobilities rise further with the increase of Cu(NH_3_)_4_^2+^ concentration because of greater complexation with the copper ion (Table 2). It also can be seen that the effective mobilities of *S*- and *rac*-atenolol are basically equal, differing slightly in the range of experiment error, and this does not change with varying Cu(NH_3_)_4_^2+^ concentrations. This suggests that the pure and mixed complexes (homo- and heterochiral associates) have largely the same effective mobility, considering that *S*-atenolol only forms a copper complex containing ligands of pure *S*-configuration via reaction (3) whereas (at least) part of *rac*-atenolol forms a mixed complex via reaction (4). Therefore, a higher effective mobility or electrodriven migration velocity of *R*-enantiomer than *S*-enantiomer in the sample segment is believed to be caused by a higher conversion ratio or higher extent of binding with copper ion, owing to the effect of high concentration of *S*-enantiomer on the equilibrium.

As shown in Figure 2B, when the amount of *R*-atenolol is increased, the step height (*H*) increases, but the step length (*l*), representing the velocity difference between the two enantiomers in sample segment, decreases because of the decline of excess *S*-enantiomer. It eventually disappears when the two enantiomers have equal content (*rac*-atenolol). This supports the above viewpoint that the difference in electrodriven migration velocity of two enantiomers relies on the effect of excess *S*-enantiomer on the binding of *R*-enantiomer with copper ion, different from the mechanism of the traditional chiral LE-CE using added enantiomerically pure chiral ligand complex as chiral selector.

## 4. Materials and Methods

### 4.1. Chemicals and Reagents

Racemic atenolol, *S*-(-)-atenolol, copper(II) sulfate pentahydrate were purchased from Sigma-Aldrich (St. Louis, MO, USA). All the chemicals used were of analytical regent grade and were used without further purification.

### 4.2. Preparation of BGE and Sample Solution

BGE solution of 4.0 mM Cu(NH_3_)_4_^2+^ in 0.2 M NH_4_OH, pH 11.0, was prepared with copper(II) sulfate pentahydrate and ammonia. The function of high concentration of ammonia in BGE was not only controlling a constant pH value but also preventing the copper ions from precipitation at high pHs. Sample solutions containing the mixtures of *S*-atenolol and *R*-atenolol with a constant total concentration of 1.0 mg∙mL^−1^ but different ratio for two enantiomers were prepared by dissolving the appropriate amount of *rac*- and *S*-atenolol standards into the BGE.

All solutions were filtered through a 0.45 μm filter prior to use.

### 4.3. Capillary Electrophoresis

A Hewlett Packard 3D CE instrument (Waldbronn, Germany) equipped with a diode array UV absorbance detector and Agilent Chemstation software Rev. A. 08.03 (847) was used for all the CE experiments. Detection was made at 230 nm with a bandwidth of 10 nm. Untreated fused silica capillaries (Polymicro, Phoenix, AZ, USA) with an internal diameter (id) of 50 μm and outer diameter (od) of 350 μm were used for separation. The length of the capillary was 60 cm, 51.5 cm to the detector. The capillary cassette temperature was set at 20 °C. New capillary was conditioned by flushing in sequence with Milli-Q water for 5 min, 1.0 M NaOH for 10 min and Milli-Q water again for 5 min at a pressure of 900 mBar. Between injections, the capillary was rinsed in sequence with Milli-Q water, 1.0 M HCl, Milli-Q water,1.0 M NaOH, Milli-Q water again and finally BGE for 2 min each at a pressure of 900 mBar. Injections were performed hydrodynamically at 50 mBar for 60 s. A separation voltage of 30 kV was used for all experiments.

## 5. Conclusions

In this work, a new approach based on SDE for the separation of atenolol enantiomers in CE was demonstrated. The copper (II) ions added into the BGE bridge the molecules of atenolol enantiomers to form homo- or heterochiral associates (complexes) that are necessary for SDE. Different distribution of the two enantiomers between the associates and single molecules, caused by the effect of high concentration of a major enantiomer on the complexation equilibrium of another enantiomer, are responsible for the difference in electrodriven migration velocities of two enantiomers. This is a completely new principle different from the traditional chiral LE-CE depending on the use of an enantiomerically pure chiral ligand complex as chiral selector. It is expected to be applicable for the analysis of other chelating chiral drugs in asymmetric synthesis and pharmaceutics.

## Figures and Tables

**Table 1:** Quantitative parameters of developed method for the analysis of *R*-atenolol content in mixtures of two enantiomers ^a^.

Calibration range	0~30% (*R*-atenolol, m/m)
Regression equation ^b^	*H =* 0.9988*C +* 1.6855
Coefficient of determination, *R*^2^	0.9989
Recovery (%)	
5% added	97.6
10% added	102.5
15% added	98.2
20% added	101.2
25% added	99.0
30% added	97.4
Repeatability (RSD, %) ^c^	
Intra-day	5.1
Inter-day	7.3

^a^ BGE and CE conditions are the same as in Figure 2. ^b^ *H*: step height (mAU); *C*: content of *R*-atenolol (%, m/m). ^c^ Value at a *R*-atenolol content of 10.0% (m/m).

**Table 2:** Electrophoretic mobilities (10^−9^ m^2^∙V^−1^∙s^−1^) of *rac*- and *S*-atenolol in BGEs of 0.2 M NH_4_OH containing different concentrations of Cu(NH_3_)_4_^2+^ with the pH fixed at 11.0.

	0	3.0 mM	4.0 mM	5.0 mM
*rac*-atenolol	0.61 ± 0.01	2.76 ± 0.01	3.06 ± 0.01	3.49 ± 0.02
*S*-atenolol	0.61 ± 0.02	2.74 ± 0.02	3.05 ± 0.01	3.51 ± 0.03

## Data Availability

Data regarding this article will be provided upon request.

## Supplementary Materials

The following supporting information can be downloaded at: https://www.mdpi.com/article/10.3390/molecules28155908/s1, Figure S1: effect of Cu(NH_3_)_4_^2+^ concentration on the step shape; Figure S2: effect of drug concentration (total concentration of two enantiomers) on the step shape; Figure S3: linear relationship between the step height on CE curve and *R*-atenolol content.
