# Supplementary material for: Copper (II) Ions Induced Self-Disproportionation of Enantiomers in Capillary Electrophoresis for the Quantification of Atenolol Enantiomers
Source: Molecules. 2023 Aug 6;28(15):5908. doi: 10.3390/molecules28155908 (PMC10420844; doi:10.3390/molecules28155908)
Supplement: Supplementary file 1 [file molecules-28-05908-s001.zip › molecules-2531589-supplementary.pdf]

Electronic Supplementary Material

Copper (II) Ions Induced Self-Disproportionation of Enantiomers in Capillary Electrophoresis for the Quantification of Atenolol Enantiomers

Shaoqiang Hu

Henan Key Laboratory of Function-Oriented Porous Materials, College of Chemistry and Chemical Engineering, Luoyang Normal University, Luoyang 471934, China; shaoqianghu@lynu.edu.cn

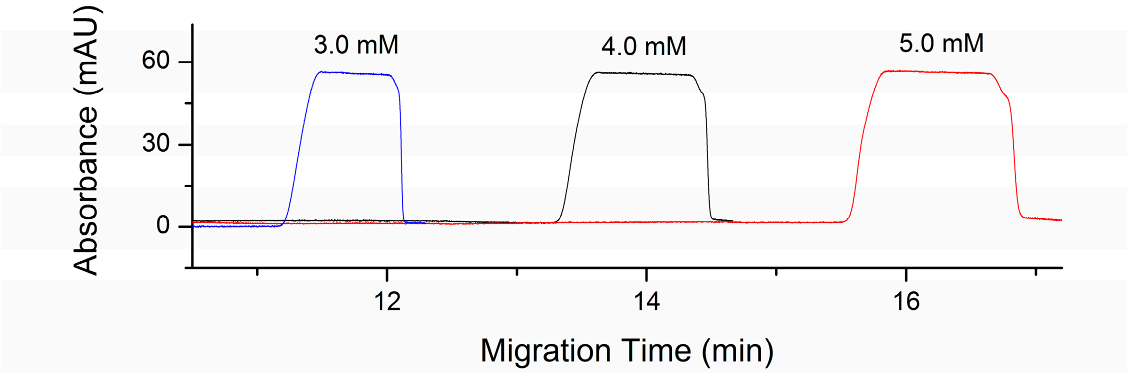

Figure S1. Effect of $Cu(NH_3)_4^{2+}$ concentration on the step shape. BGE component besides $Cu(NH_3)_4^{2+}$: 0.2 M NH_4OH, pH 11.0. Sample solution: mixtures of *S*- and *R*-atenolol in BGE with a total concentration of 0.4 mg·mL^{-1} and *R*-atenolol content of 10.0% (m/m). Capillary: id 50 µm, od 350 µm, length 60.0 cm, 51.5 cm to detector. Capillary temperature: 20 °C. Hydrodynamic injection at 50 mBar for 60 s. Separation voltage: 30 kV. Detection wavelength: 230 nm.

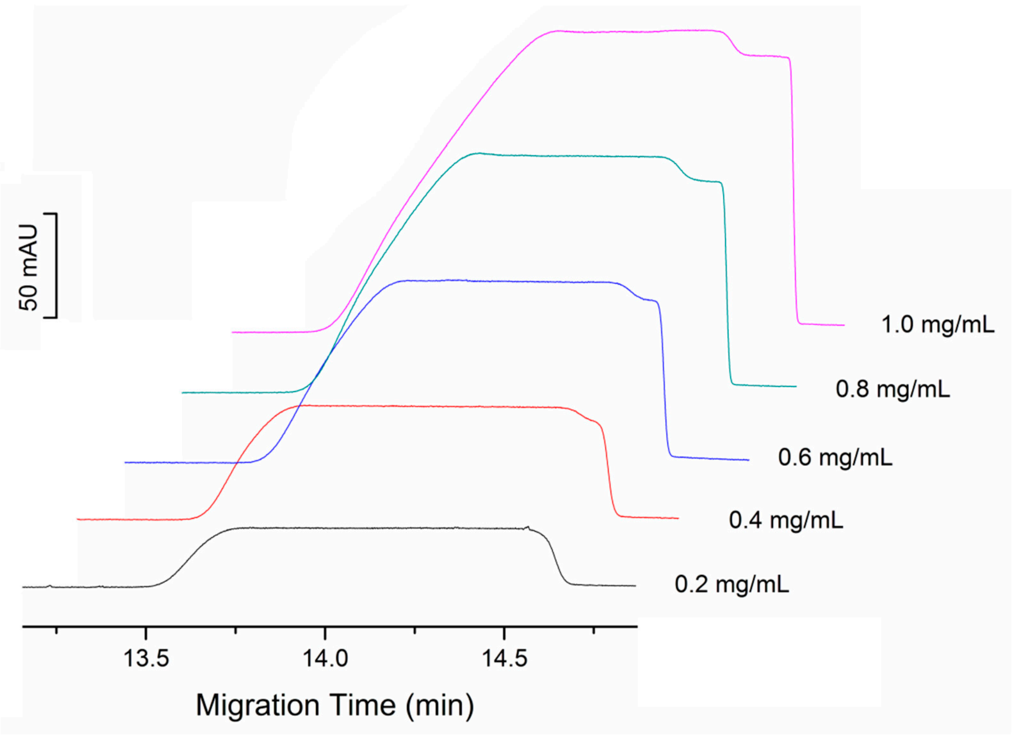

Figure S2. Effect of drug concentration (total concentration of two enantiomers) on the step shape. Sample solutions: mixtures of *S*- and *R*-atenolol in BGE with different total concentrations but a fixed *R*-atenolol content of 10.0% (m/m). BGE: 4.0 mM $Cu(NH_3)_4^{2+}$ in 0.2 M NH_4OH, pH 11.0. CE conditions are the same as in Figure S1.

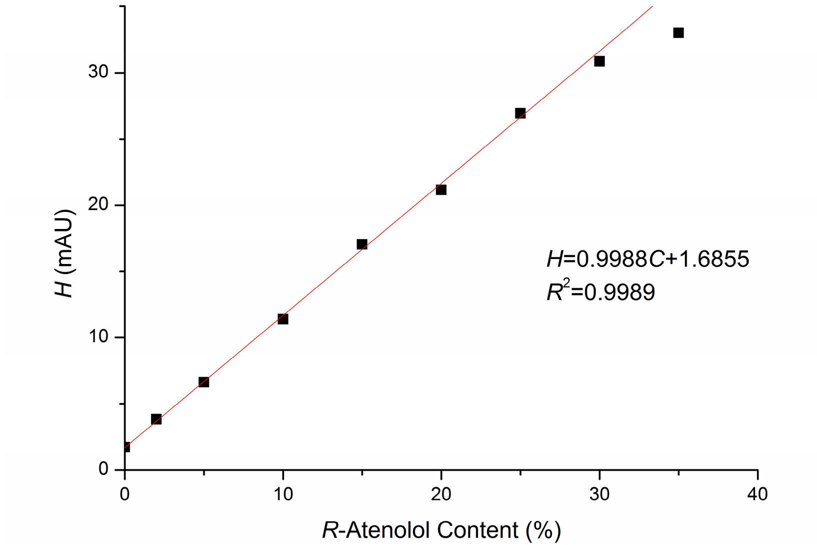

Figure S3. Linear relationship between the step height on CE curve and *R*-atenolol content. Sample solutions: mixtures of *S*- and *R*-atenolol in BGE with a fixed total concentration of 1.0 mg·mL^{-1} but different *R*-atenolol contents (m/m). BGE and CE conditions are the same as in Figure 2.
